# Graphene-Based Wireless Tube-Shaped Pressure Sensor for In Vivo Blood Pressure Monitoring

**DOI:** 10.3390/mi10020139

**Published:** 2019-02-20

**Authors:** Nagisa Inoue, Yoshihiko Koya, Norihisa Miki, Hiroaki Onoe

**Affiliations:** School of Integrated Design Engineering, Graduate School of Science and Technology, Keio University, 3-14-1 Hiyoshi, Kohoku, Yokohama, Kanagawa 223-8522, Japan; n_inoue@keio.jp (N.I.); yoshik817@a8.keio.jp (Y.K.); miki@mech.keio.ac.jp (N.M.)

**Keywords:** pressure sensor, graphene, wireless, implantable device, tube

## Abstract

We propose a wireless pressure sensor composed of a graphene sheet and a transmitter coil integrated with a polydimethylsiloxane (PDMS) tube. The pressure inside the tube was monitored wirelessly using an external receiver coil. We then monitored the typical blood pressure range, 12–20 kPa, using this fabricated sensor by changing the turn number of the receiver coil and the overlapping length of the coils. Furthermore, we demonstrated wireless blood pressure measurement by connecting our sensor to the blood vessel of a rat. Our results suggested that this sensor can be easily inserted between an implantable medical device and blood vessels for in vivo blood pressure monitoring. The proposed wireless pressure sensor could also be suitable for monitoring in vivo implanted medical systems, such as artificial organs and pump systems.

## 1. Introduction

Wearable medical devices and artificial organs have been advanced significantly with the aim of assisting the daily lives of patients who require continuous clinical treatment [1,2,3,4,5,6,7]. Wearable insulin pumps [8] and wearable dialysis systems [9] are the most popular examples and have been widely used for the treatment of patients in practical settings. Such external wearable medical systems usually have external pumps that connect to in vivo blood vessels by tubes through the skin. The use of such tubes can lead to peritonitis and aggravation, in addition to limiting the patients’ daily activities. In recent years, to avoid the above problems and improve the quality of life (QOL) of patients, implantable medical devices that do not require connections between the inside and outside of the body have been developed, such as the artificial heart [10,11], artificial kidneys [12], dialysis systems [13], and insulin pumps [14]. In these systems, as the flow of bodily fluids or drugs is controlled by the implanted pump systems, the avoidance of a pump failure or thromboembolism is essential to prevent the development of serious conditions [15]. Thus, the monitoring of the fluid flow around the implanted devices is essential.

Generally, various wireless monitoring sensors have been adopted to monitor the pressure of fluids in implantable systems in vivo [16]. For example, pressure sensors and flow sensors are incorporated in stents [17] and grafts [18], respectively. However, the implanted systems containing the integrated medical devices with monitoring sensors tend to be complicated and expensive, as it is necessary to develop a specially designed monitoring sensor system for each device. It is therefore preferable to have a low-cost, simple, and space-saving wireless monitoring sensor that can be externally attached to various implantable devices independently.

**Figure 1:**
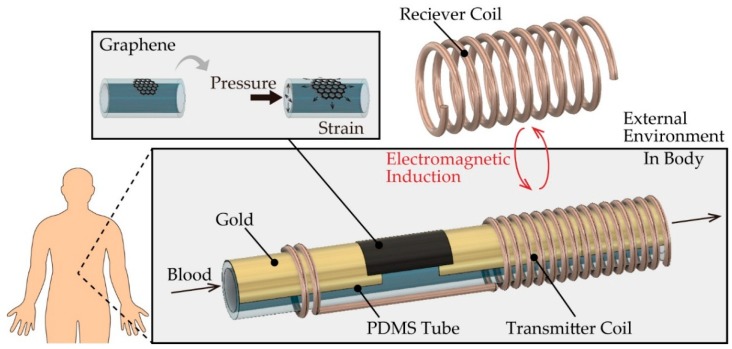
Conceptual illustration of the graphene-based wireless tube-shaped pressure sensor.

Here, we propose a wireless tube-shaped pressure sensor for in vivo blood pressure monitoring that can be easily attached to the various device (Figure 1). This sensor is composed of a graphene-based inline pressure sensor [19] and a coil wound up to the tube as a transmitter. The advantage of the use of graphene is that graphene could be transferred to a curved surface [20] as a sensing element because it is extremely thin [21]. This wireless pressure sensor can be easily inserted between the implanted device and a blood vessel due to its construction on a microfluidic elastic tube. An external receiver coil can then wirelessly monitor the applied pressure inside the tube through electromagnetic inductive coupling. In addition, the use of an internal power supply is unnecessary for our monitoring sensor. In this study, we also evaluate the performance of the wireless tube-shaped pressure sensor and demonstrate that the pulse pressure in blood vessels can be monitored wirelessly using our proposed sensor.

## 2. Principle and Design 

**Figure 2:**
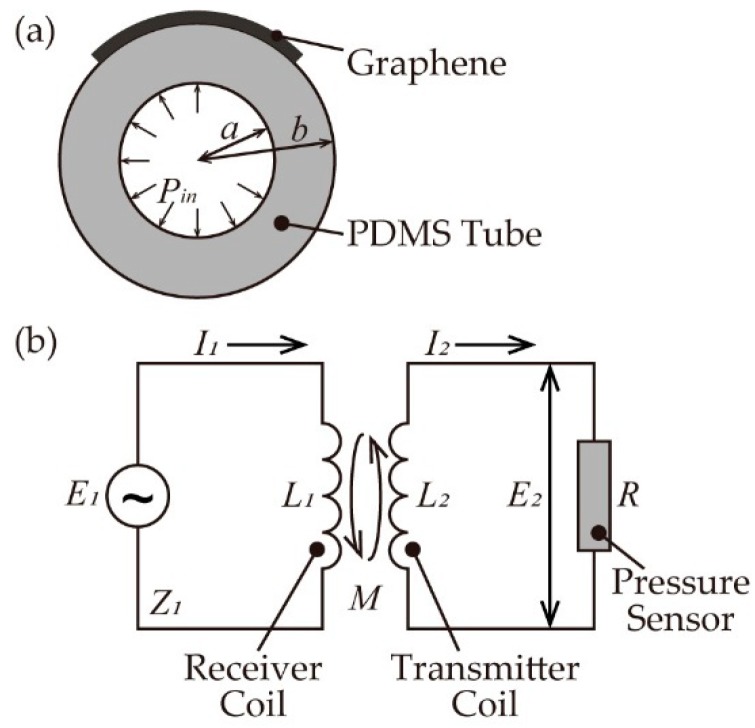
The principle of our proposed sensor. (**a**) Schematic cross-sectional diagram. (**b**) Schematic circuit diagram.

A schematic cross-sectional drawing of the proposed sensor is shown in Figure 2a. As the internal pressure in an elastic tube can be measured from the change in electrical resistance of the graphene [19], the relationship between the sensitivity of the sensor and the pressure is given by

(1) $$\frac{\Delta R}{R}=\frac{2}{E}\cdot \frac{a^{2}}{b^{2}-a^{2}}\cdot GF\cdot P_{in}$$

where the electrical resistance of graphene is *R*, the change in the electrical resistance of graphene is Δ*R*, *E* is the Young’s modulus of the tube, *a* and *b* are the inner and outer diameters of the tube, respectively, *P_in_* is the gauge pressure, and *GF* is the gauge factor. Equation (1) shows that the sensitivity of the sensor can be tuned by modifying the dimensions of the fabricated tube and the properties of the polydimethylsiloxane (PDMS).

A schematic circuit diagram of the proposed sensor and receiver coil is shown in Figure 2b. Upon electromagnetic induction between the coils, the change in the electric resistance of the graphene alters the impedance of the receiver coil’s circuit, which can in turn be used to measure the pressure. As the sensitivity of the impedance depends on the inductance, the coil distance, and the angles of the coils., we theoretically estimated the relationship between the impedance of the receiver coil’s circuit and the resistance of the sensor [22]. The voltage *E*_1_ and *E*_2_ of each circuit is given by

(2) $$E_{1}=j\omega L_{1}I_{1}\pm j\omega MI_{2},$$

(3) $$E_{2}=j\omega L_{2}I_{2}\pm j\omega MI_{1}=-RI_{2},$$

where *I*_1_ and *I*_2_ are the currents of each circuit, *L*_1_ and *L*_2_ are the inductance of the transmitter coil and the receiver coil, respectively, and *M* is the mutual inductance between two circuits. The sign of *M* depends on the direction of the current and the magnetic flux. From Ohm’s law and Equations (2) and (3), the impedance of the receiver coil *Z*_1_ can be represented by

(4) $$Z_{1}=\frac{E_{1}}{I_{1}}=j\omega L_{1}\mp \frac{\omega^{2}M^{2}}{j\omega L_{2}+R}.$$

The mutual inductance *M* is defined by $M=k\sqrt{L_{1}L_{2}}$, where *k* is the coupling coefficient. Equation (4) shows that the impedance of the receiver coil, *Z*_1_, changes according to differences in the resistance value of graphene, *R*, and the changing ratio of the impedance increases according to the inductance of the receiver coil and the increase in the coupling coefficient. In addition, the inductance *L* is defined by $L=K\mu SN^{2}/l$ [23], where *K* is the self-inductance of the finite-length solenoid relative to that of an infinite solenoid, *μ* is the magnetic permeability, *S* is the cross-sectional area of the coil, *N* is the number of turns, *l* is the length of the coil, and the coupling coefficient *k* is expressed as k=f(d,θ), where *d* and *θ* are the distance and the angle of the coil, respectively.

In this paper, the diameter of the tube was set to the typical vessel diameter of the renal artery (i.e., 4 mm) [24], and the tube thickness was set at 1 mm for monitoring in the normal blood pressure range of 12–20 kPa [25].

## 3. Experimental Methods

### 3.1. Fabrication of the PDMS Tube and Wiring

**Figure 3:**
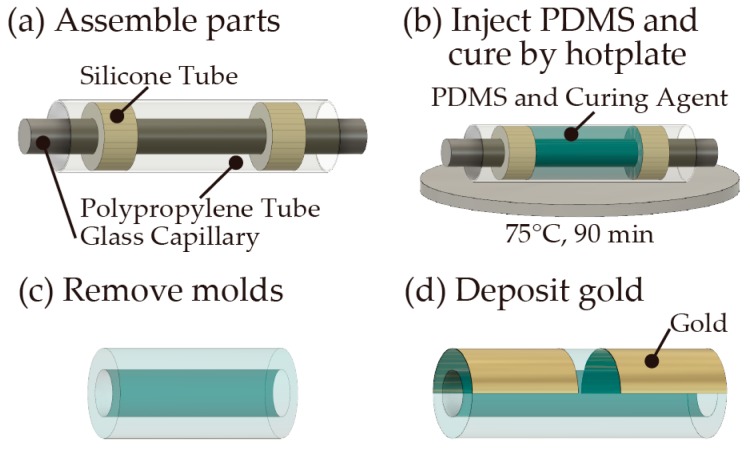
The polydimethylsiloxane (PDMS) tube molding and wiring processes.

We initially molded the PDMS tube [19] using a glass capillary (*ϕ* = 3 mm) (NARISHIGE, Tokyo, Japan, G3) as the inner template and a polypropylene tube (*ϕ* = 4 mm) (Daiso Sangyo, Hiroshima, Japan) as an outer template. PDMS is a suitable material for adjusting the tube dimensions and stiffness by changing the mold dimensions and the mixing ratio with a curing agent. The inner template was inserted into the outer template and fixed at its center using a silicone tube spacer (AS ONE, Osaka, Japan, 9-869-04) (Figure 3a). Following assembly of the mold, the mixture (10:1 *w/w*) of the PDMS base polymer solution and the curing agent (Dow Corning, Midland, MI, USA, Silpot184) was injected into the mold (Figure 3b) and the injected PDMS prepolymer was degassed and cured using a hotplate at 75 °C for 90 min. After that, the mold was removed to obtain the PDMS tube (Figure 3c), of which a cross-sectional image was obtained using a digital microscope (KEYENCE, Osaka, Japan, VH-5500) to evaluate the inner and outer diameters. Finally, a gold film (approximately 3 μm) was deposited on the surface of the tube as wiring using a vapor deposition apparatus (Vacuum Device, Ibaraki, Japan, VE2012) (Figure 3d).

### 3.2. Transfer of the Graphene Sheet and Coil Winding

**Figure 4:**
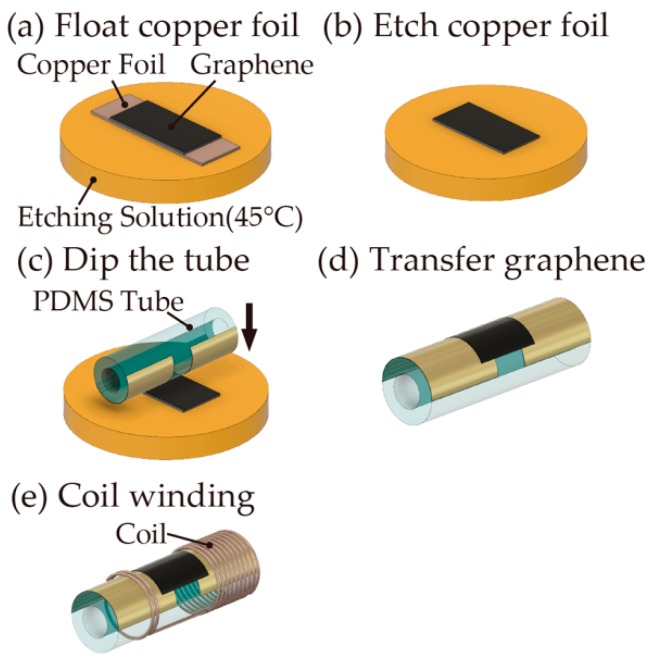
The graphene transfer and coil winding processes.

A multilayered graphene sheet with 10–20 nm (30–50 layers) (Graphene Platform Corp., Tokyo, Japan) was transferred to the fabricated PDMS tube according to the literature [20]. The multilayered graphene sheet (2.0 mm × 5.0 mm) formed on the copper foil was then floated on a Cu etching solution (Sunhayato Corp., Tokyo, Japan, H-1000A) at 45 °C (Figure 4a) to achieve the etching of the copper foil (Figure 4b). The graphene floating on the solution was picked up by the PDMS tube (Figure 4c), and the graphene sheet was firmly adhered to the surface of the PDMS tube through van der Waals forces (Figure 4d). The surface of the tube was then washed with deionized water, and a 75-turn coil was assembled with an enameled copper wire (KYOWA HARMONET, Kyoto, Japan, 2UEW0.26 mm) around the PDMS tube (Figure 4e). As a receiver coil, an enameled copper wire (KYOWA HARMONET, Kyoto, Japan, 2UEW0.5 mm) was wound up to a diameter of 15 mm with 25, 50, and 75 turns.

### 3.3. Pressure Measurement by the Fabricated Pressure Sensor

The pressure applied inside the PDMS tube was measured as follows. One side of the tube was connected to a syringe that was controlled by a syringe pump (KD Scientific, Holliston, MA, USA, LEGATO 180) to apply air pressure inside the tube. The other side of the tube was connected to a reference pressure sensor (KEYENCE, Osaka, Japan, AP-V180) to monitor the pressure. To evaluate the resistance change of the transferred graphene, a digital source meter (Keithley, Solon, OH, USA, 2614B) was employed. For the wireless measurements, the receiver coil was set close to the fabricated sensor, and the impedance of the receiver coil was measured using an LCR meter (Hioki, Nagano, Japan, IM3536).

### 3.4. Pressure Measurement In Vivo

All animals used in the experiments received human care. The experimental protocol was approved by the Laboratory Animal Care and Use Committee following Keio University guidelines. A female Sprague-Dawley rats (age 32–36 weeks; Clea Japan, Co., Tokyo, Japan) was used. The rat was anesthetized with Isoflurane inhalation solution (Pfizer Japan, Tokyo, Japan). After that, the limbs were fixed to the surgical table with adhesive tape, and the abdominal cavity was opened. A catheter was inserted into the femoral artery and femoral vein, and a wireless pressure sensor was connected to the catheter. The wireless pressure sensor was fabricated on a silicone tube with an inner diameter of 0.5 mm and an outer diameter of 1.0 mm according to the size of the blood vessel of the rat. Blood pressure was monitored wirelessly using a receiving coil with a diameter of 3 mm and 30 turns.

## 4. Results

### 4.1. Fabricated Pressure Sensor

**Figure 5:**
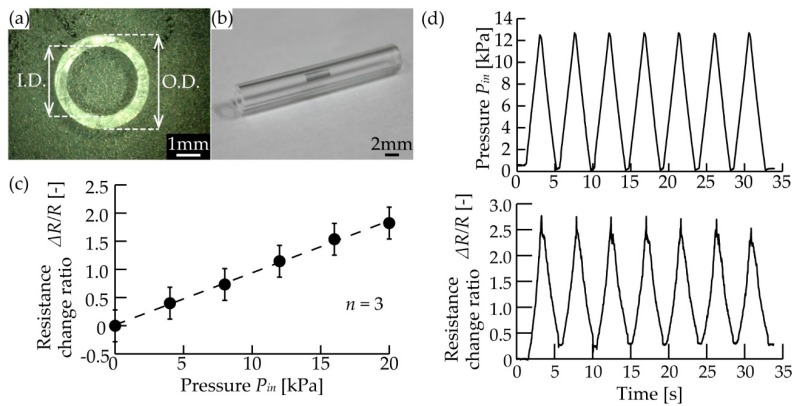
The fabricated pressure sensor. (**a**) Cross-sectional image of the PDMS tube. (**b**) Photograph of the PDMS tube with the transferred graphene. (**c**) The relationship between the applied pressure and the resistance change ratio of transferred graphene with three independent sensors. Error bars indicate standard deviation (*n* = 3). (**d**) The relationship between the applied cyclic pressure and the resistance change ratio of the transferred graphene.

We fabricated the PDMS tube using a mold with an inner diameter of 3 mm and an outer diameter of 1 mm. As mentioned in the previous research [19], the diameter of the tube affects the sensitivity of the sensor. The dimensions of the mold and the fabricated tube are shown in Table 1, and a cross-sectional image is shown in Figure 5a. The PDMS tube was fabricated with a relative error of <1% for the designed mold, and the standard deviations of the measured diameters were <0.077 (*n* = 5).

We measured the change in the electrical resistance, *R*, of the graphene transferred to the PDMS tube (Figure 5b) by applying a positive air pressure using a syringe and monitoring the pressure with the reference pressure sensor. Figure 5c shows the relationship between the applied pressure, *P_in_*, and the changing ratio of resistance, Δ*R/R* (*n* = 3). The changing ratio of electrical resistance, Δ*R/R*, increased from 0 to 1.84 in the pressure range of 0–18 kPa and a sensitivity of 0.10 kPa^−1^ was achieved. In addition, Figure 5d shows the resistance when 12 kPa of pressure was applied to the sensor in seven equal cycles. The resistance varied in the same cycle following the pressure between 2–7 kPa.

### 4.2. Wireless Pressure Measurements

**Figure 6:**
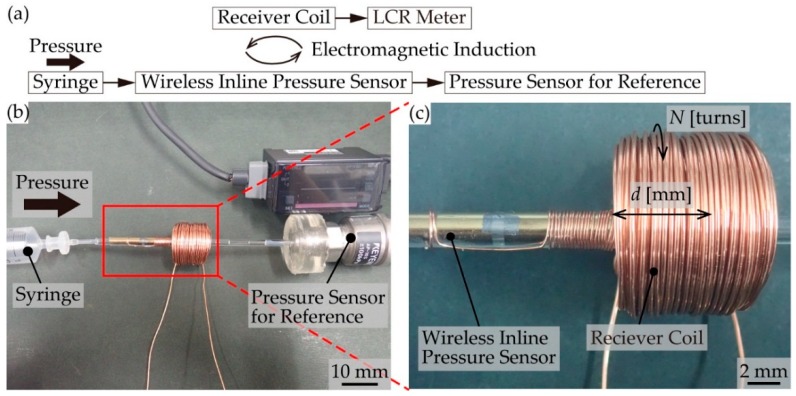
The measurement setup. (**a**) A schematic diagram. (**b**) A photographic image. (**c**) The fabricated wireless inline pressure sensor.

The proposed pressure sensor converts the pressure inside the tube, *P_in_*, according to the changes in the impedance of the receiver coil, *Z*_1_, through a change in the resistance of graphene, *R*. We evaluated the changes in the impedance of the receiver coil, *Z*_1_, by applying an air pressure, *P_in_,* using a syringe. The pressure inside the tube, *P_in_*, was monitored using the reference pressure sensor (Figure 6a,b). In addition, we also evaluated the effect of the number of receiver coil turns, *N*, and the overlapping length of the coils, *d*, on the changing ratio of the impedance, Δ*Z*/*Z* (Figure 6c). Here, we defined the sensor response as Δ*Z*/*Z*. The average contact resistance between the gold and graphene was 33.7 Ω, and the standard deviation was 12.5 Ω (*n* = 3).

**Figure 7:**
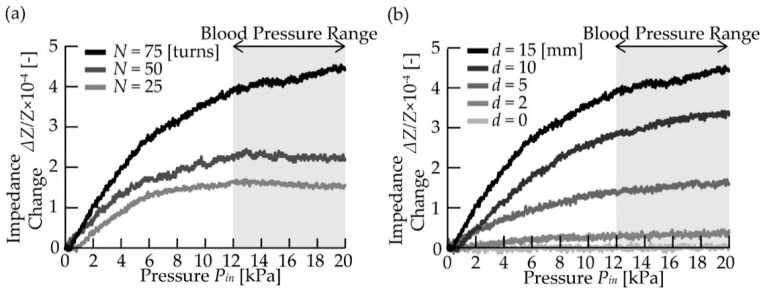
Wireless pressure measurements for evaluating sensor performance. (**a**) The relationship between the applied pressure and the impedance of the receiver coil with different turns. (**b**) The relationship between the applied pressure and the impedance of the receiver coil with different overlapping lengths.

Figure 7a shows the relationship between the applied pressure, *P_in_*, and the changing ratio of the impedance of the receiver coil, Δ*Z*/*Z*, for different numbers of coil turns, *N*. The pressure, *P_in_*, was applied as a ramp function at (*P*(t) = 0.5 [kPa/s] × *t* [s]), and the changing ratio of the impedance, *ΔZ/Z*, increased with the pressure range of 0–20 kPa. In addition, the value of Δ*Z*/*Z* increased as the number of coil turns, *N,* increased. Furthermore, the sensor with the largest number of turns (*N* = 75 turns) exhibited the highest response in terms of sensitivity (Δ*Z*/*Z* = 4.4 × 10^−4^ at 20 kPa) and that with the smallest number of turns (*N* = 25 turns) exhibited the lowest response (Δ*Z*/*Z* = 1.4 × 10^−4^ at 20 kPa), as expected from Equation (4). 

Figure 7b shows the relationship between the applied pressure (0–20 kPa), *P_in_*, and the changing ratio of the impedance, Δ*Z*/*Z*, for the different overlapping coil lengths, *d*. As indicated, the value of Δ*Z*/*Z* increased upon increasing *d*, with a sensor response of Δ*Z*/*Z* = 4.4 × 10^−4^ at 20 kPa being obtained for *d* = 15 mm. In contrast, in the absence of overlap (*d* = 0 mm), no change in the impedance was observed. This was attributed to the fact that the mutual inductance, *M*, increases as the overlapping coil length, *d*, increases, which in turn increases the changing ratio of the impedance, Δ*Z*/*Z*, as indicated in Equation (4). These results indicate that highly sensitive blood pressure measurement could be carried out by increasing the number of turns of the receiver coil, *N*, and the overlapping coil length, *d*.

### 4.3. Pressure Measurement In Vivo

**Figure 8:**
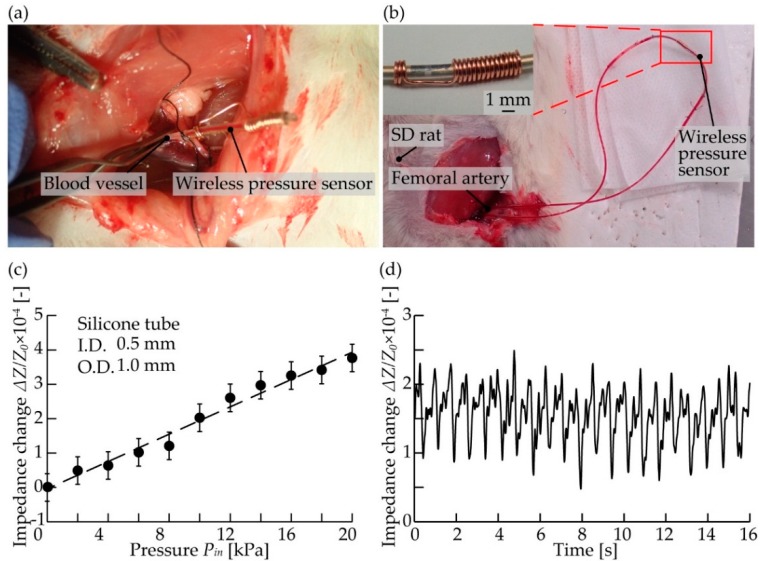
In vivo blood pressure monitoring. (**a**) A wireless pressure sensor connected to the femoral artery. (**b**) Setup for measuring blood pressure. (**c**) The relationship between impedance change and applied pressure. (**d**) Obtained signals of impedance change versus time in blood pressure measurement.

As a demonstration of our sensor, we performed wireless blood pressure monitoring in vivo. We confirmed that a sensor with a diameter of 1.0 mm could be directly inserted into a blood vessel (femoral artery) of a rat (Figure 8a). For blood pressure monitoring, our sensor was connected to the femoral artery via a catheter (Figure 8b). In order to confirm the performance of our sensor, the relationship between the impedance change of the receiver coil and the pressure was evaluated in vitro (Figure 8c). The sensor response, Δ*Z*/*Z*, was approximately 4.0 × 10^−4^ at 20 kPa. The blood pressure was monitored wirelessly by the receiving coil and impulse response was obtained from the impedance change of the receiving coil (Figure 8d). The impedance change varied from 1.0 × 10^−4^ to 1.5 × 10^−4^, corresponding to a pulse pressure of 5.0 to 7.5 kPa estimated by the sensor response in Figure 8c. Previous literature has shown that the pulse pressure of an SD rat was 5.5 kPa (standard deviation 1.3 kPa) [26], which is reasonably close to our measured value.

## 5. Discussion

We demonstrated an attachment-type pressure sensor for an implantable medical device. The sensor response changed with the applied pressure, and a changing ratio of the impedance, Δ*Z*/*Z*, of 4.4 × 10^−4^ was obtained at 20 kPa in the case of the sensor with a large number of receiver coil turns (*N* = 75 turns) and the longest overlapping length (*d* = 15 mm). This result indicates that the proposed sensor could be employed to wirelessly measure an inner pressure in the context of blood pressure monitoring for an implantable medical device. We also found that the sensor could be adjusted to various dimensions and sensitivities, thereby demonstrating its versatility for application in various devices. In addition, this sensor is expected to be widely applicable due to its simple structure, easy fabrication, and low cost.

In this study, we applied a transmitter coil and a graphene sheet as the pressure-sensing element to an elastic tube and succeeded in wirelessly monitoring the applied pressure through the measurement of the impedance changes. However, there remain additional improvements to be developed prior to its practical use. For example, as the graphene sheet responds to the bending of the elastic tube, the sensor signal may be disturbed by body movements. To overcome this problem, the sensor requires a protective cover that prevents the elastic tube from bending when the sensor is used in vivo. In addition, the positional relationship between the transmitter coil and the receiver coil also affects the strength of the response. By developing a wearable-type receiver coil [27], the position between the receiver and transmitter coils could be expected to be fixed, keeping the obtained signal more suitable for practical settings. Following such improvements, we except that our proposed pressure sensor could be applied to artificial kidneys [12], dialysis systems [13], and insulin pumps [14] to monitor the device performance by attaching our sensor to the upstream or downstream of the implanted medical device.

Our proposed sensor may also be suitable for measuring pumping pressures in microfluidic systems due to its small size and facile attachment. In particular, in recent years, the field of in vitro tissue engineering has attracted drug testing and regenerative medicine [28], and the perfusion and fluid shear stress of the in vitro reconstructed tissues have been recognized to be important parameters for tissue maturation [29]. It therefore appears possible to monitor the applied fluid pressure by inserting our sensor between the pumps and the in vitro cultured tissues without disturbing the culture environment. We envisage that our wireless pressure sensor could become a simple, easily-attachable, and space-saving measuring tool for application in various pressure monitoring situations.

## 6. Conclusions

We proposed a wireless tube-shaped pressure sensor for in vivo blood pressure monitoring. This sensor was able to wirelessly measure pressure by integrating graphene and a transmitter coil with a polydimethylsiloxane (PDMS) tube. The changing ratio of the impedance, Δ*Z*/*Z*, was found to be 4.4 × 10^−4^ for a pressure of 20 kPa in the case of the receiver coil with the largest number of turns and the longest overlapping coil length. By connecting the sensor to the rat femoral artery, a pulse pressure of 5.0–7.5 kPa was obtained. We therefore believe that our pressure sensor will be applicable for monitoring the pressure within various in vivo implantable devices.

## Figures and Tables

**Table 1:** Dimensions of the mold design and the fabricated tube.

	Mold Design	Fabricated Tube
Inner Diameter (I.D.) (mm)	3.00	2.98
Outer Diameter (O.D) (mm)	4.00	3.96
